# Robust Fractional Low Order Adaptive Linear Chirplet Transform and Its Application to Fault Analysis

**DOI:** 10.3390/e27070742

**Published:** 2025-07-11

**Authors:** Junbo Long, Changshou Deng, Haibin Wang, Youxue Zhou

**Affiliations:** 1 College of Electronic Information Engineering, Jiujiang University, No. 551, Qianjin East Road, Jiujiang 332000, China; ljb829@jju.edu.cn; 2 College of Computer and Big Data Science, Jiujiang University, No. 551, Qianjin East Road, Jiujiang 332000, China; 6120062@jju.edu.cn (H.W.); 6120043@jju.edu.cn (Y.Z.)

**Keywords:** adaptive chirplet transform, infinite variance process, *α* stable distribution, Gaussian colored noise

## Abstract

Time-frequency analysis (TFA) technology is an important tool for analyzing non-Gaussian mechanical fault vibration signals. In the complex background of infinite variance process noise and Gaussian colored noise, it is difficult for traditional methods to obtain the highly concentrated time-frequency representation (TFR) of fault vibration signals. Based on the insensitive property of fractional low-order statistics for infinite variance and Gaussian processes, robust fractional lower order adaptive linear chirplet transform (FLOACT) and fractional lower order adaptive scaling chirplet transform (FLOASCT) methods are proposed to suppress the mixed complex noise in this paper. The calculation steps and processes of the algorithms are summarized and deduced in detail. The experimental simulation results show that the improved FLOACT and FLOASCT methods have good effects on multi-component signals with short frequency intervals in the time-frequency domain and even cross-frequency trajectories in the strong impulse background noise environment. Finally, the proposed methods are applied to the feature analysis and extraction of the mechanical outer race fault vibration signals in complex background environments, and the results show that they have good estimation accuracy and effectiveness in lower MSNR, which indicate their robustness and adaptability.

## 1. Introduction

The frequency of a non-stationary signal is a one-dimensional non-fixed function relative to the time variable, and the TFA method is an effective analysis technology for it. The traditional time-frequency technologies, such as the linear class STFT [1], the bilinear class PWVD [2], the scale class CWT transformation [3], and the parameter model ARMA class [4] are affected by the Heisenberg Gaber uncertainty principle, cross-term interference, etc., which lead to a large calculation volume, low time-frequency concentration, and poor anti-interference ability. Therefore, the research on improving the time-frequency energy concentration has become a current hotspot. Post-processing time-frequency techniques usually perform synchronous compression or extraction operations to improve the time-frequency concentration of the obtained TFR. To address the problem of poor time-frequency effect for chirp signals, Wei Deyun et al. proposed a generalized synchrosqueezing S-transform and improved the multi-spectra synchrosqueezing transform by fusing multiple TFRs of the signals [5]. Tu Qiyu et al. proposed the local maximum multisynchrosqueezing transform algorithm by combining multiple synchronous compression techniques and using iterative redistribution to aggregate fuzzy energy in the original S-transform TFR, which has certain advantages in dealing with stress frequency and multi-component signals [6]. Correia et al. improved time-frequency resolution of the original STFT result by combining the generalized adaptive polynomial window function and synchrosqueezing technology [7]. For strong time-varying instantaneous frequency signals, a generalized synchrosqueezing transform was proposed in [8], which reconstructs the original TFR by constructing a generalized synchrosqueezing operator GSSO. Xiong, Hongqiang proposed a synchrosqueezing generalized phase-shifting S-transform method and applied it to ground-penetrating radar tunnel detection, which has better advantages in terms of strong focusing, accuracy, and anti-interference ability [9]. In order to enhance the applicability of TFR, Chen Yumeng et al. extended synchrosqueezing to the two-dimensional level and proposed a two-dimensional synchrosqueezing transform [10].

Although SST and SET can improve the time-frequency resolution, the performance of such methods completely depends on the concentration degree of the original TFR adopted. For this reason, the kernel matching-based chirplet transform time-frequency technique was born. The LCT method is an efficient time-frequency technique that is only limited by the matching of frequency components in linear signals [11,12,13]. The improved algorithm based on chirplet transformation has better adaptability to a certain extent [11], and general linear chirplet transform [12] and velocity synchronous linear chirplet transform [13] were subsequently proposed. Recently, in order to achieve higher resolution and accuracy in ultrafast measurement technology, an optical chirplet transform was proposed in [14]. Aiming at the drawback that SCT cannot match multi-component signals, Chen Hui et al. constructed a multi-vector based on the synchrosqueezing chirplet transform and proposed a synchronous spline-kernelled chirplet squeezing transform to improve the concentration of TFR [15]. Yuan PingPing introduced the spline chirplet transform into local maximum synchrosqueezing and combined it with linear chirplet transform and proposed a local maximum synchrosqueezing spline chirplet transform, which has good precision and stability for signals with noise [16]. Subsequently, they addressed the issue of low accuracy of the instantaneous frequency of the structure and proposed an improved multi-synchrosqueezing spline-kernelled chirplet transform based on the three-parameter Gaussian window function and multi-synchrosqueezing compression technology to improve the accuracy and practicality of the method [17]. Wang Jianguo et al. introduced an innovative synchronous redistribution operator into the LMSST algorithm and proposed a local maximum synchrosqueezing reassigning chirplet transform to improve the reliability of the algorithm [18].

Aiming at multi-component signals with cross-frequency trajectories in the time-frequency domain, Yunpeng Guan et al. proposed an adaptive linear chirp transform (ALCT) [19], which first uses a series of chirp rates to calculate the LCT. Then, the real-time frequency of the signal is adaptively extracted through iteration and removal. ALCT avoids the problem of frequency estimation error caused by modal mixing and eliminates the mutual interference trouble caused by the cross-frequencies of multi-component signals. To further reduce the mixing error problem of multi-component signals in the overlapping area, an enhanced ALCT (EALCT) method was proposed [20], which utilizes the ridge path regrouping (RPRG) algorithm to separate the real-time frequency set and reconstruct each component signal. Zhu Yan et al. refined the ALCT TFR of the signal to obtain higher resolution and energy concentration based on the synchronous extraction technology [21]. For multi-component signals with short frequency intervals and strong background noise, SBCT matches the real-time frequencies of multi-component signals by changing the length of the basis function, thereby obtaining a higher time-frequency energy concentration of the signal [22]. Hou Yating et al. improved SBCT using the local maximum synchrosqueezing technique and proposed a local maximum synchrosqueezing SBCT algorithm to obtain better time-frequency energy concentration and more accurate instantaneous frequency trajectory [23]. Kaur combined a hybrid parallel-series attention-based deep learning network with SBCT to extract feature information from images, which can effectively improve the classification accuracy [24]. Zhao Dezun et al. constructed a synchronous reassignment operator to adaptively extract the maximum value of the real-time frequency from the SBCT of the signal and reconstruct its TFR, which can obtain higher time-frequency concentration results [25]. Subsequently, they made algorithm improvements based on SBCT and redistributed the amplitude coefficients of the SBCT results by using the synchronous extrusion technique [26]. The local optimal TFR is extracted by constructing the local optimal theory based on Renyi entropy, and then the ideal time-frequency amplitude is obtained from the time-frequency curve based on the local maximum value extraction criterion [27]. By combining SBCT, a real-time fault detection technology for edge devices based on adaboost is proposed, which can effectively improve the detection accuracy and reduce the delay [28].

The time-frequency techniques discussed above mainly focus on how to improve the concentration of the TFR of the signal. However, most of them provide prior knowledge of the signal and assume that the results are obtained under Gaussian weak background noise. In a complex working background environment, there exists a strong impulse process α stable distribution noise. Because it has no finite variance, it is often called an infinite variance process [29,30,31]. Under the background noise of the infinite variance process, the functions of the time-frequency technology discussed above deteriorate or even fail. Therefore, we must seek improved methods that are suitable for the environments. Aiming at the environments with α stable distribution noise, a forward-looking orthogonal matching pursuit algorithm for sound vector sensor arrays based on fractional-order statistics was proposed to enhance the estimation accuracy of arrival estimation [32]. Based on STFT and fractional low-order statistics, the multiple window function was used in [33], and according to this, a fractional low-order multiple window was proposed for mechanical fault signal analysis. Based on the synchroextracting transform and fractional statistics, Fractional low order multi-synchrosqueezing transform and fractional low order second-order synchroextracting transform were proposed in [34].

For multi-component signals with crossover frequency trajectories in a time-frequency domain under an infinite variance process environment, we introduce fractional lower-order statistics and extend it with FLOCT instead of the CT method and propose a robust fractional low-order adaptive linear chirplet transform (FLOALCT) algorithm. Meanwhile, in order to make the high-concentration SBCT TFR method have wider applicability, we have extended it by the same means and proposed a resilient and generalized fractional low-order adaptive scaling proportional basis function linear frequency-modulated wavelet transform new method (FLOASCT). The FLOALCT and FLOASCT methods exhibit better TFR performance without prior conditions of noise type and intensity and have good adaptability. In the strong impulse infinite variance process noise environment, both methods show significant advantages compared with the existing methods, especially when the characteristic index parameter α<1, as the performance advantages of the improved algorithm are more obvious.

The structure of this paper is as follows. The novel statistical model and the actual fault vibration signal are introduced in Section 2. The FLOALCT and FLOASCT methods are derived in Section 3, and the calculation steps and processes of the algorithms are summarized. In Section 4, through the time-frequency analysis and fault feature extraction simulations of the actual fault vibration signals, the superiority of the proposed FLOALCT and FLOASCT algorithms is discussed and demonstrated. Finally, the conclusion is summarized in Section 5.

## 2. Novel Statistical Model for the Mechanical Vibration Signals

### 2.1. Novel Statistical Model

**Figure 1:**
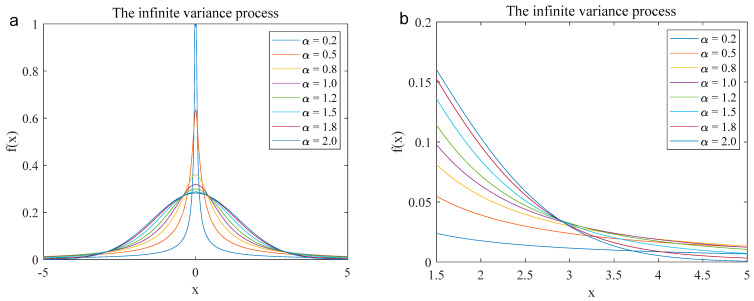
The PDFs waveform of the infinite variance process in different characteristic index parameters (*α* = 0.2, 0.5, 0.8, 1.0, 1.2, 1.5, 1.8, and 2.0). ((**a**). Complete waveform (from −5 to 5); (**b**). Local waveform (from 1.5 to 5)).

The infinite variance process, also known as the α stable distribution process, is named so because it does not have a finite second moment. This process can be characterized by its characteristic functions:

(1) $$\phi (t)=\exp\left\{j\mu t-\gamma {\left|t\right|}^{\alpha }[1+j\beta sign(t)\omega (\tau ,\alpha )]\right\}$$

where α, β, μ, and γ are the characteristic index, symmetry parameter, position parameter, and dispersion coefficient, respectively. Here, α, β, μ, and γ represent the characteristic exponent, skewness parameter, location parameter, and scale parameter, respectively. Figure 1 shows the probability density functions (PDFs) of the infinite variance process under various conditions: α = 0.2, 0.5, 0.8, 1.0, 1.2, 1.5, 1.8, and 2.0. From the figures, we can know that in the Gaussian process α=2, when x=5, f(x) converges completely, and its value approaches 0. However, PDFs of lower-order infinite variance processes (α<2) converge more slowly and exhibit long tails, and the smaller the characteristic index value α is, the more severe the tailing will be.

### 2.2. Novel Statistical Model for the Mechanical Vibration Signals

**Figure 2:**
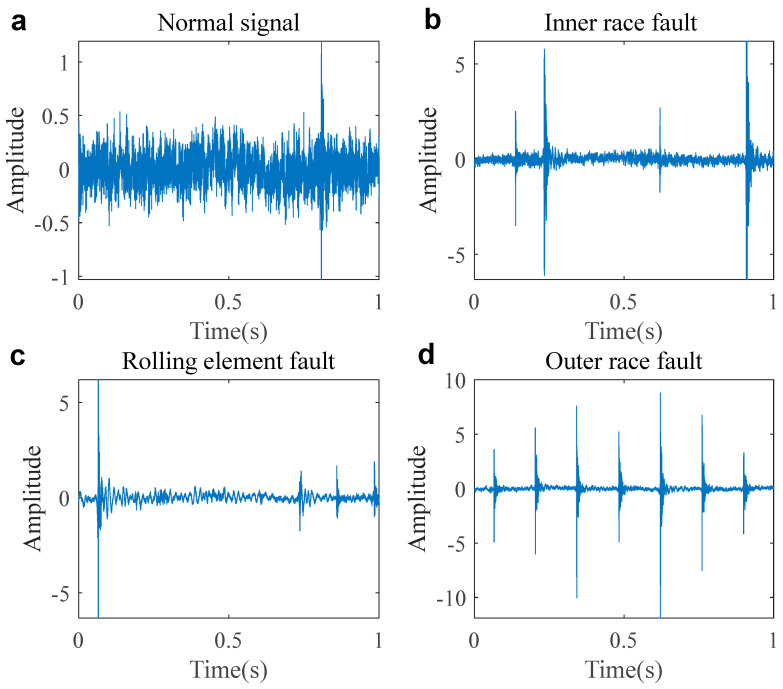
The waveform of the normal and fault signals. ((**a**). Normal signal; (**b**). inner race fault; (**c**). rolling element fault; (**d**) outer race fault).

In this section, the real mechanical vibration experimental signals come from the Jiangnan University bearing dataset [35]. The dataset includes normal mechanical vibration signals, as well as fault signals for the outer race, inner race, and rolling elements. The rotational speed condition of the dataset is 800 revolutions per minute (rpm), with a sampling frequency of 50,000 Hz. Figure 2 shows the waveforms of the normal and fault signals. A novel statistical model, the α stable distribution, is used to simulate the mechanical vibration signals, and their parameters are obtained, as shown in Table 1. From the table, we can see that except for the normal signal’s parameter α=2, the other fault signal α values are less than 2, proving that they indeed belong to the infinite variance α stable distribution process.

**Figure 3:**
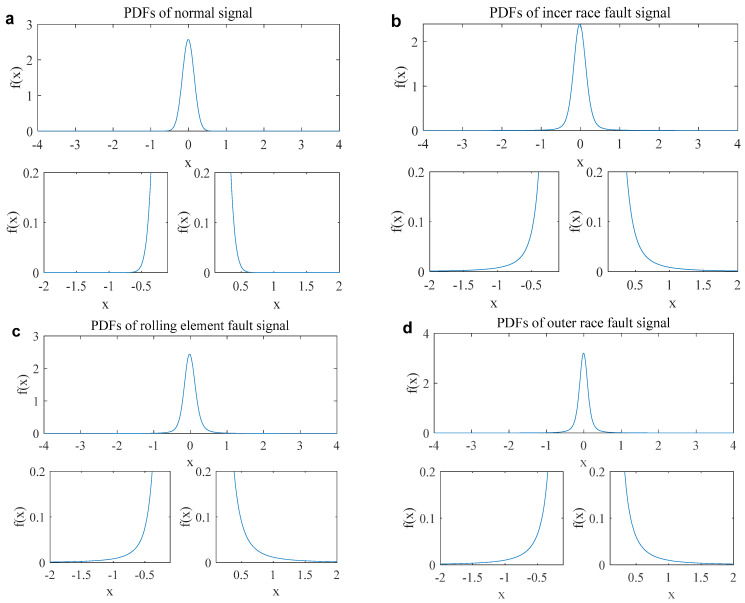
PDFs of the normal and fault signals. ((**a**). Normal signal; (**b**). inner race fault signal; (**c**). rolling element fault signal; (**d**). outer race fault signal).

Figure 3 shows the probability density function (PDF) of the normal and fault signals. The PDF of the normal signal in Figure 3a exhibits Gaussian distribution characteristics and converges normally. However, the PDFs of the inner race, rolling element, and outer race fault signals have long tails and slow convergence, displaying typical α stable distribution characteristics, as shown in Figure 3b–d.

## 3. Robust FLOALCT Time Frequency Representation

### 3.1. FLOALCT TFR Method

#### 3.1.1. Principle

The Linear Chirplet Transform (LCT) introduces a kernel function based on the Short-Time Fourier Transform (STFT). LCT is actually the energy peak distribution of a group of chirplets with different chirp rates in the time-frequency domain, and it is also a linear transformation, suitable for the superposition calculation of multiple signals. Based on the definition of LCT, we introduce fractional low-order cumulants and define the fractional lower order liner chirplet of an analytic signal considering time-varying instantaneous frequencies transform (FLOLCT) as

(2) $$FLOLCT(u,\omega ,c)=\int_{-\infty }^{+\infty }s^{<p>}(t+u)h(t)\Psi^{*}(t,\omega ,c)dt=\int_{-\infty }^{+\infty }\bar{s}\Psi^{*}(t,\omega ,c)dt$$

where $\bar{s}=s^{<p>}(t+u)h(t)$ is the fractional lower-order statistic of the windowing function. <p> represents the p-order moment of s(t+u), where p is the real coefficient, α is the characteristic index of α stable distribution, and when s(t+u) is a real signal, $s^{<p>}(t+u)={\left|s(t+u)\right|}^{p-1}\cdot sign[s(t+u)]$, α<p≤2. However, if it is a complex signal, $s^{<p>}(t+u)={\left|s(t+u)\right|}^{p+1}/s^{*}(t+u)$, 0<p≤α/2, and ∗ represents the complex conjugation. h(t) is unit amplitude and the normalized real window function expressed as

(3) $$h(t)=\frac{1}{\sqrt{2\pi }\sigma }e^{-\frac{1}{2}{\left(\frac{t}{\sigma }\right)}^{2}}$$

Because the window function h(t) is normalized, its integral over the entire time span is equal to 1.

(4) $$\Psi (t,\omega ,c)={\left(e^{-\frac{1}{2}jct^{2}}e^{-j\omega t}\right)}^{*}=e^{j(ct+\omega )t}=e^{j\int_{-\infty }^{+\infty }2\pi f_{basis}(t)dt}$$

where ∗ represents the complex conjugation, Ψ(t,ω,c) represents a set of complex waves with frequency varying linearly over time, and $f_{basis}(t)$ represents the frequencies of the bases. When s(t+u)=1, then $\bar{s}=s^{<p>}(t+u)h(t)=h(t)$, Equation (2) can be written by

(5) $$\Gamma (\omega ,c)=FLOLCT(u,\omega ,c){|}_{s=1}=\int_{-\infty }^{+\infty }h(t)\Psi^{*}(t,\omega ,c)dt$$

Based on the Ville and fractional lower moments theory, the p-order moment of a non-stationary analytic signal $s(t)=A(u)e^{j\varphi (t)}$ can be written as

(6) $$\begin{array}{c} s^{<p>}(t)=A(t)e^{j\int \omega (t)dt}=A(t)e^{j\varphi (t)}\\ =\frac{{\left|A(t)\right|}^{p+1}}{{\left(A(t)e^{j\phi (t)}\right)}^{*}}=\frac{{\left|A(t)\right|}^{p+1}}{A(t)}e^{j\phi (t)}=A^{p}(t)e^{j\phi (t)} \end{array}$$

where A(u) is its amplitude of the signal s(t), and φ(t) and ω(t) are its phase and the instantaneous frequency, respectively. In a short time window u, according to Taylor expansion, IF ω(t) can be approximated as ${\left.\omega (t)\right|}_{t\in u}\approx \omega (u)+\omega^{\prime }(u)(t-u)$. ω(u) is IF of the signal s(t) at time u, and $\omega^{\prime }(u)$ is the first derivative of ω(u) with respect to time u, then $\omega^{\prime }(u)=d\omega (u)/du$, which represents the IF change rate.

Substituting IF ω(t) into Equation (2), the amplitude of the signal s(t) at time u and frequency ω(u) and instantaneous chirp rate c(u) employing the FLOLCT method can be expressed as

(7) $$\begin{array}{c} \bar{FLOLCT(u,\omega ,c)}=\left|FLOLCT(u,\omega ,c)\right|=\left|\int_{-\infty }^{+\infty }s^{<p>}(t-u)h(t)\Psi^{*}(t,\omega (u),c(u))dt\right|\\ =\left|\int_{-\infty }^{+\infty }A^{p}(t)e^{j\varphi }e^{j\left[\omega (u)t+\frac{1}{2}\omega^{\prime }(u)t^{2}\right]}h(t)e^{-j\left[\omega (u)t+\frac{1}{2}c(u)t^{2}\right]}dt\right|\\ =\left|\int_{-\infty }^{+\infty }A^{p}(t)e^{j\varphi (t)}e^{j\frac{1}{2}\left[\omega^{\prime }(u)-c(u)\right]t^{2}}h(t)dt\right| \end{array}$$

Since the window function has been normalized, $\int_{-\infty }^{+\infty }h(t)dt=0$. When $\omega^{\prime }(u)=c(u)$, the amplitude of FLOLCT of the signal s(t) gets the maximum, then

(8) $$\bar{FLOLCT(u,\omega ,c)}\leq \left|\int_{-\infty }^{+\infty }A^{p}(t)e^{j\varphi (t)}h(t)dt\right|=A^{p}(t)$$

From Equation (8), the best arguments $c^{\prime }(u)$ can be gotten by

(9) $$c^{\prime }(u)=\arg\underset{c}{\max}\bar{FLOLCT(u,\omega ,c)}$$

Hence, the FLOLCTTFR of the signal s(t) employing the ALCT and multi-synchrosqueezing transform method in [19,36] can be written as

(10) $$\begin{array}{c} FLOLCTTFR(t,\omega )=\int_{-\infty }^{+\infty }FLOLCT(u,\omega ,c^{\prime }(t))\delta (\omega -\omega (u))d\omega \\ =A^{p}(u)e^{j\varphi (u)}\delta (\omega -\omega (u)) \end{array}$$

According to the method, the signal $s^{<p>}(t)$ can be reconstructed employing the signal reconstruction algorithm in [34], written by

(11) $$x^{<p>}(t)=\frac{1}{2\pi h(0)}\int_{-\infty }^{+\infty }FLOLCTTFR(t,\omega )d\omega =\frac{A^{p}(u)e^{j\varphi (u)}\delta (\omega -\omega (u))}{2\pi h(0)}$$

where h(0) is the amplitude of the window function at time t=0. Then the signal s(t) can be expressed as

(12) $$x(t)=\frac{A^{1-p}(u)}{2\pi h(0)}\int_{-\infty }^{+\infty }FLOLCTTFR(t,\omega )d\omega =\frac{A(u)e^{j\varphi (u)}\delta (\omega -\omega (u))}{2\pi h(0)}$$

The FLOALCT time-frequency method is designed for N(N≥1) multi-component signals $s(t)=\sum_{n=1}^{N}s_{n}(t)$ based on the aforementioned FLOLCT, which is a method for iteratively extracting time-frequency signals. First, the signal s(t) is substituted into the FLOLCT formula in Equation (2), and then

(13) $$FLOLCT(u,\omega ,c)=\sum_{n=1}^{N}FLOLCT_{n}(u,\omega ,c)$$

where $FLOLCT_{n}(u,\omega ,c)$ represents the FLOLCT of the *n*-th signal. By searching and extracting the peak values in the entire FLOLCT time-frequency domain of s(t), we can obtain the optimal arguments $c_{n}^{\prime }(u)$ for the n-th signal, which is expressed as

(14) $$c_{n}^{\prime }(u)=\arg\underset{c}{\max}\bar{FLOLCT(u,\omega ,c)}$$

Therefore, the time-frequency reconstruction of the n-th signal can be represented as

(15) $$FLOLCTTFR_{n}^{\prime }(t,\omega )=\int_{-\infty }^{+\infty }FLOLCT(u,\omega ,c_{n}^{\prime }(t))\delta (\omega -\omega_{n}(u))d\omega$$

By removing the time-frequency distribution $FLOLCTTFR_{n}^{\prime }(t,\omega )$ of the n-th signal in the FLOLCT TF domain, the FLOLCT TFR of the remaining n−1 signals is obtained as follows:

(16) $$FLOLCTTFR_{n-1}^{\prime }(t,\omega )=FLOLCT(u,\omega ,c)-FLOLCTTFR_{n}^{\prime }(t,\omega )$$

Then, by cyclically executing the iterative process of Equations (14)–(16), the individual time-frequency components of n signals can be obtained. Finally, by time-frequency aggregation of each time-frequency component of the multi-component signal s(t), the FLOALCT TFR of the signal can be obtained.

FLOALCT is achieved by sorting the signals in the FLOLCT time-frequency domain according to their amplitudes. Firstly, the first component with the largest amplitude is evaluated and eliminated. Then, the next amplitude sorting is performed, and the component with the largest amplitude is evaluated again. Through this continuous descending iterative estimation, the time-frequency independent components of all signals can be obtained. Finally, based on the characteristics of the independent time-frequency components of the signal, FLOLCTTFR is used to reconstruct the TFR of the signal.

The calculation steps and processes of the Algorithm 1 FLOALCT algorithm are summarized as follows:
**Algorithm 1.** FLOALCT algorithm for multi-component signals1: Initialization phase and parameter setting. Including normalized window and p-order moment parameter p et al.
2: Calculate fractional p-order moment of the signal s(t).
3: Chirplet basis generation. Including chirp rate range, time axis for window and chirplet kernels. 4: Iterative component extraction: set initial residual $r_{0}(t)=s(t)$, k=1. 5: While ${TFR}_{x_{k}}$ > threshold. 6:      Compute FLOLCT of residual $r_{k}(t)$ using Formula (2). 7:      Search for the peak in the time-frequency domain and extract the optimal parameter
$c_{k}^{\prime }(u)$ of the *k*-th component using Formula (14). 8:      Generate TFR of the *k*-th component ${TFR}_{x_{k}}(t,f)$ using Formula (15).
9:       Compute reconstructed component. 10:     Update residual ${TFR}_{res}^{\left(k\right)}={TFR}_{res}^{\left(k-1\right)}-{TFR}_{x_{k}}$.
11:     n = n + 1.
12:     End. 13: Reconstruct the *k*-th component
$s_{k}(t)$ of the original signal using Formulas (11) and (12).
14: Combine all components using Formula (13).

When c=0, FLOLCT degenerates to FLOSTFT. For complex signals, when p=1 in Equation (2), FLOLCT degenerates to the LCT method, and FLOALCT becomes the ALCT algorithm. Therefore, FLOALCT is an extension of the ALCT algorithm. It is a generalized ALCT that can operate stably in environments with complex infinite variance process noise and Gaussian mixture noise. FLOALCT can effectively avoid the adverse effects of strong components on weak components in infinite variance process environment and sequentially and adaptively iteratively separate the independent time-frequency components of the signal. Moreover, it is not necessary to adopt the relevant noise reduction program for the infinite variance process in advance. The program can be directly run to obtain the FLOALCT time-frequency distribution of the signal in a complex background noise environment.

#### 3.1.2. Application Review

In this section, the test signal consisting of two components is used to evaluate the performance of the proposed FLOALCT and the exiting ALCT methods, which is given as

(17) $$s(t)=\sin\left[2\pi (50t+20\sin(2t))\right]+\sin\left[2\pi (2+9t)t\right]=s_{1}(t)+s_{2}(t)$$

**Figure 4:**
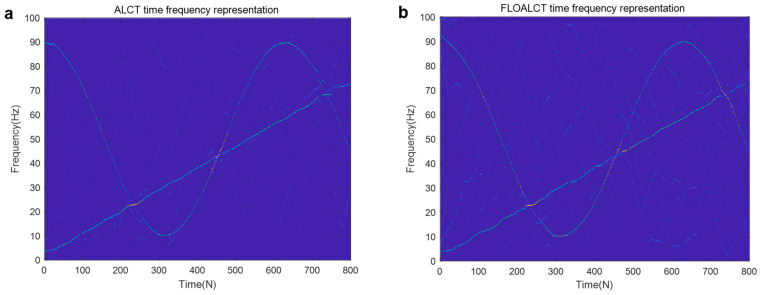
TFR of s(t) under Gaussian noise environment (α=2, SNR=8 dB). ((**a**). ALCT method; (**b**). FLOALCT method).

**Figure 5:**
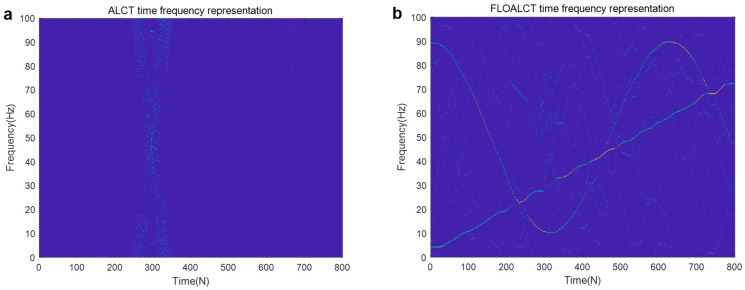
TFR of s(t) in infinite variance process noise environment (α=0.8, MSNR=18 dB). ((**a**). ALCT method; (**b**). FLOALCT method).

The sampling frequency of the signal s(t) is set to 200 hz, and its instantaneous frequency trajectory, which is a nonlinear function of time, can be repressed as f(t)=(50+40cos(2t))+(2+18t), where $s_{1}(t)$ is with IF law 50+40cos(2t), and IF of $s_{2}(t)$ is (2+18t). The FLOALCT and ALCT time frequency representation methods are applied to reveal a nonlinear change in the frequency of the signal s(t) with time. The experiments are conducted with infinite variance process noise and Gaussian noise separately, and the results are shown in Figure 4 and Figure 5, respectively, among which SNR is used for Gaussian noise and mixed signal noise ratio (MSNR), which is given by $MSNR=10\log_{10}(\sigma_{s}^{2}/\gamma )$. $\sigma_{s}$ is with variance law, and γ is the dispersion coefficient of infinite variance process noise.

**Figure 6:**
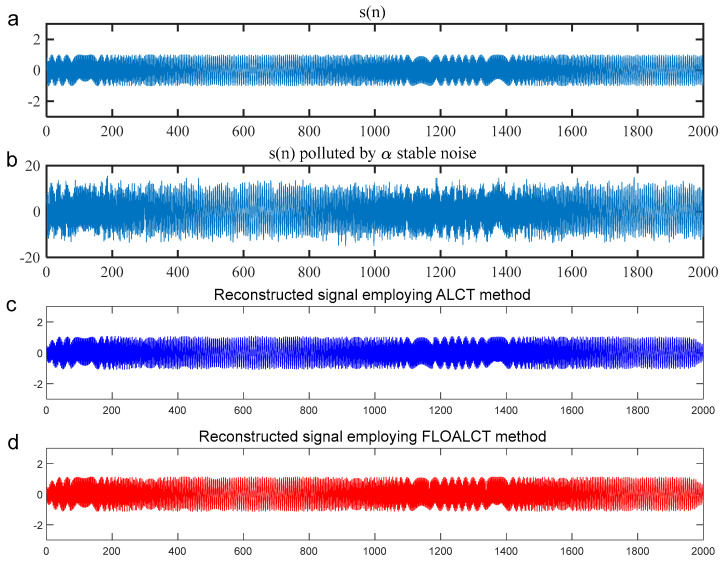
(**a**) The experimental original signal s(n); (**b**) the experimental original signal polluted by Gaussian noise (SNR=8 dB); (**c**) the signal reconstructed by the ALCT method; (**d**) the signal reconstructed by the improved FLOALCT method.

**Figure 7:**
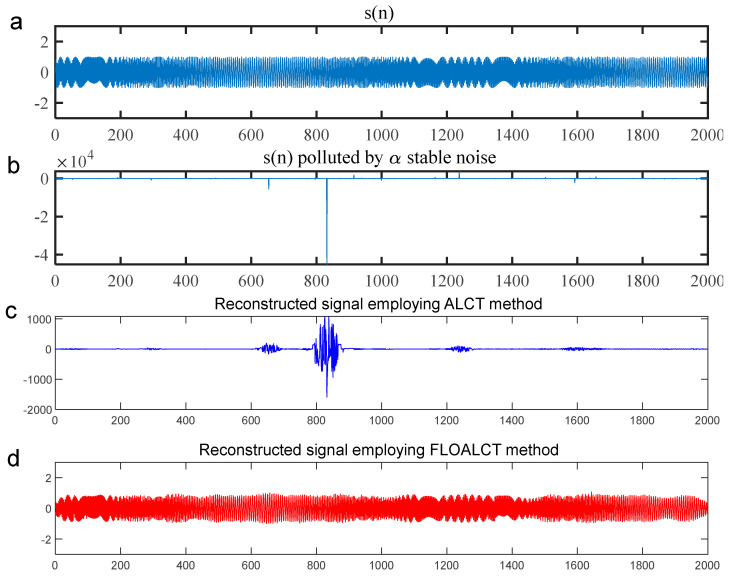
(**a**) The experimental original signal s(n); (**b**) the experimental original signal polluted by infinite variance process noise (α=0.8, MSNR=18 dB); (**c**) the signal reconstructed by the ALCT method; (**d**) the signal reconstructed by the improved FLOALCT method.

To better test the signal time-frequency reconstruction capability of the FLOALCT algorithm, the mixed mean square error (MSE) of the experimental original signal s(t) and the reconstructed signal $\hat{s}(t)$ employing the ALCT and FLOALCT methods can be given by

(18) $$MSE=10\log_{10}\left\{\frac{1}{K}\sum_{k=1}^{K}{\left[\hat{s}(t)-s(t)\right]}^{2}\right\}$$

where K is the number of the Monte-Carlo experiment. The reconstruction experiments with the ALCT and FLOALCT methods are conducted with infinite variance process noise and Gaussian noise separately, and the results are shown in Figure 6 and Figure 7, respectively.

**Figure 8:**
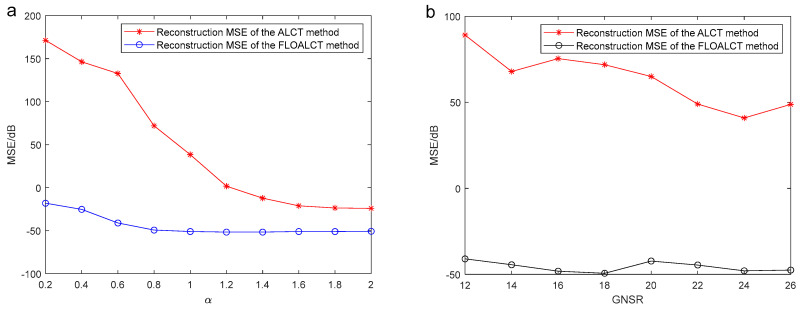
(**a**) Reconstruction MSE of the ALCT and FLOALCT methods under different characteristic index α (0.2–2) when MSNR=18 dB; (**b**) reconstruction MSE of the ALCT and FLOALCT methods under different MSNR (12 dB–26 dB) when α=0.8.

In following, we give the FLOALCT method applied in analyzing the reconstruction MSE of the experimental original signal s(t), compared with the ALCT method under different characteristic index α and MSNR. The simulation result is shown in Figure 8.

#### 3.1.3. Remarks

It can be seen that when α=2, and SNR=8 dB, infinite variance process noise is degenerated to Gaussian noise. The frequency resolution of the proposed FLOALCT and the existing ALCT methods can be easily determined from Figure 4a,b. Both methods have nearly the same effect, and the frequency change of the signal s(t) is clearly visible with time. In the case of infinite variance process noise, the signal is severely contaminated by strong impulsive noise, the ALCT method almost loses its ability, and the time-frequency image of the signal in Figure 5a is completely false. The proposed FLOALCT algorithm can almost ignore the influence of strong pulses. Because of the introduction of fractional lower moments, it is still effective and can better display the time-frequency variation image of the signal, as show in Figure 5b. It reflects the capability advantage of the improved FLOALCT in the time-frequency analysis of signals in harsh environments.

For the result in Figure 6, Figure 6a gives the original signal s(t), and it is heavily polluted by Gaussian noise in Figure 6b (SNR=8 dB). Both ALCT and FLOALCT methods provide a satisfactory signal time-frequency reconstruction capability, as shown in Figure 6c and Figure 6d, respectively. It is demonstrated that the two methods are less affected by Gaussian noise. However, the result in Figure 7b show that the original signal s(t) is engulfed by infinite variance process noise (α=0.8, MSNR=18 dB), and only the pulse process can be seen. However, the experimental original signal s(t) cannot be reconstructed by the ALCT method due to the interference of infinite variance process noise in Figure 7c. The FLOALCT method still shows a satisfactory reconstruction result, which proves the toughness of the improved algorithm.

Figure 8a illustrates the reconstruction mean square error (MSE) variation of both the ALCT and FLOALCT methods with different characteristic index values of α, ranging from 2 to 3, when MSNR=18 dB. It can be seen from the figure that when α changes from 1.2 to 2, the MSE of the two methods is not much different, but that of the FLOALCT method is always lower. When α<1.2, due to the influence of impulse noise, the reconstruction MSE of the ALCT increases rapidly. However, FLOALCT’s remains stable with little change. It reflects the ability of FLOALCT to resist low characteristic index impulse noise. The reconstruction MSE variation of the ALCT and FLOALCT methods under different MSNR values (12 dB–26 dB) is shown in Figure 8b. When α=0.8, the reconstruction MSE of the ALCT is higher than 40 dB, which indicates that the reconstructed signal differs significantly from the original signal. FLOALCT gives a relatively low reconstruction error, which varies slightly around −40 dB, and provides a superior signal reconstruction result over ALCT.

### 3.2. FLOASCT TFR Method

#### 3.2.1. Principle

Based on the definition of FLOLCT in Equation (2), we can express it in an alternative form as follows:

(19) $$FLOLCT(u,f,t)=\int_{-\infty }^{+\infty }s^{<p>}(t)h(t-u)\Psi^{*}(u,f,t)dt=\int_{-\infty }^{+\infty }\bar{s}\Psi^{*}(u,f,t)dt$$

where $\bar{s}=s^{<p>}(t)h(t-u)$ is fractional lower-order statistics with a window function. h(t) is a Gaussian window function, which is given in Equation (2). Ψ(t,f,u) in Equation (19) can be written as follows:

(20) $$\Psi (u,f,t)={\left(e^{-j2\pi ft}e^{-j\pi c{\left(t-u\right)}^{2}}\right)}^{*}={\left(e^{-j2\pi (ft+\frac{1}{2}c{\left(t-u\right)}^{2})}\right)}^{*}={\left(e^{-j2\pi \varphi (u,f,t)}\right)}^{*}=e^{j2\pi \varphi (u,f,t)}$$

where $\varphi (u,f,t)=ft+\frac{1}{2}c{\left(t-u\right)}^{2}$ is a phase function, and ∗ represents the complex conjugation. In Equation (20), f corresponds to the center of the signal frequency, and u represents the center of time. The parameter c is a constant known as the chirp rate. The real real-time frequency trajectory matching of the signal in the FLOLCT time-frequency domain is achieved by changing the wavelet basis through its rotation, which is the second-order derivative of φ(u,f,t) with respect to time t, which can be expressed by

(21) $$c=\varphi^{\prime \prime }(u,f,t)=d\varphi^{\prime }(u,f,t)/dt=d(f+c(t-u))/dt=-\arctan\theta$$

where θ represents the rotating angle; its ridge tangent is expected to be as close to the slope value of the IF trajectory as possible. This approach ensures that the frequency-modulated wavelet is closely matched with the intermediate frequency trajectory value, thereby achieving higher energy concentration in the FLOLCT time-frequency representation.

Due to its constant chirp rate, FLOLCT is unable to achieve high energy concentration at each time point when processing nonlinear frequency-modulated signals, which presents certain limitations in analyzing such signals. To address this issue, we introduce a new kernel phase function based on the idea of the SBCT algorithm, enabling the modulation frequency to adaptively follow the transformation according to the frequency component of the real-time point and the center time, thereby enabling the scaling of the original basis to achieve the matching target. This kernel phase function can be expressed as follows:

(22) $$\varphi_{s}(u,f,t,a_{1},a_{2},\cdots a_{k})=ft+\sum_{k=1}^{K}fa_{k}{\left(t-u\right)}^{k+1}$$

where the parameter set $a_{1},a_{2},\cdots a_{k}$ adaptively varies according to the frequency and center time of the FLOLCT time-frequency points. By substituting $\varphi_{s}(u,f,t,a_{1},a_{2},\cdots a_{k})$ for φ(u,f,t) in Equation (21), we can get fractional lower order adaptive scaling chirplet transform (FLOASCT) of the analytic signal s(n), which can be written as follows:

(23) $$FLOASCT(u,f,t)=\int_{-\infty }^{+\infty }s^{<p>}(t)h(t-u)\Phi^{*}(u,f,t)dt=\int_{-\infty }^{+\infty }\bar{s}\Phi^{*}(u,f,t)dt$$

(24) $$\Phi (u,f,t)=e^{j2\pi \varphi_{s}(u,f,t,a_{1},a_{2},\cdots a_{k})}$$

By performing a second-order derivative of $\varphi_{s}(u,f,t,a_{1},a_{2},\cdots a_{k})$ in Equation (22) with respect to time t, we can obtain the adaptive chirp rate c(u,f,t), which can be written by

(25) $$\begin{array}{c} c(u,f,t)=\varphi_{s}^{\prime \prime }(u,f,t,a_{1},a_{2},\cdots a_{k})=d\varphi_{s}^{\prime }(u,f,t,a_{1},a_{2},\cdots a_{k})/dt\\ =d(f+\sum_{k=1}^{K}(k+1)fa_{k}{\left(t-u\right)}^{k})/dt\\ =f\sum_{k=1}^{K}k(k+1)a_{k}{\left(t-u\right)}^{k-1}\\ =2fa_{1}+f\sum_{k=2}^{K}k(k+1)a_{k}{\left(t-u\right)}^{k-1}=-\arctan\theta \end{array}$$

For any point in the FLOASCT time-frequency domain, when t=u, the adaptive chirp rate $c(u,f,t)=2fa_{1}$, and the rotating angle $\theta =\arctan(-2fa_{1})$. Unlike the constant rotating angle θ in FLOLCT, the FLOASCT algorithm adjusts the rotating angle to match the changes in instantaneous frequency f. For example, if there are N frequency components in a multi-component signal, then the corresponding N rotating angles may be written as

(26) $$\theta_{n}=-\arctan(2f_{n}a_{1}),\;n=1,2,3,\cdots ,N$$

We consider a small amount of time variation Δt centered around the time point, and t=u+Δt. Then, the rotating angles $\theta_{n}$ in Equation (25) for every frequency center can be expressed by

(27) $$\theta_{n}=-\arctan\left(f_{n}\sum_{k=1}^{K}k(k+1)a_{k}{\left(\Delta t\right)}^{k-1}\right),\;n=1,2,3,\cdots ,N$$

As long as the adaptive parameters $a_{1},a_{2},\cdots a_{k}$ in $\varphi_{s}(u,f,t,a_{1},a_{2},\cdots a_{k})$ are appropriately set, the chirp rate c can closely match the slope of the instantaneous frequency, so as to achieve the true real-time frequency trajectory matching of the FLOASCT time-frequency domain signal. The calculation steps and processes of the Algorithm 2 FLOASCT algorithm are summarized as follows:
**Algorithm 2.** FLOASCT algorithm1: Initialization including p-order moment parameter p, time vector t, frequency center point f_center, angle candidate set angle_candidates, et al. 2: Calculate fractional p-order moment of the signal s(t) using Formula (6).
3: Sliding window processing.
4: Multi-angle candidate.
5:      For theta in angle candidates:
6:      Calculate $a_{1}$–$a_{k}$: $a_{1}$ = −tan(theta)/(2*mean(f_center)). 7:      Optimize parameters $a_{2}$–$a_{k}$ (Gradient descent method).
8:      Calculate adaptive chirp rate.
9:      End for. 10: Construct kernel function Φ(u,f,t) according to Formula (24).
11: Calculate the energy concentration index of each candidate angle.
12: Calculate the sub-FLOASCTTFR using Formula (23).
13: Select the optimal TFR.
14: Output final time-frequency representation.

When the chirp rate c=0, it becomes parallel to the time axis, and thus FLOLCT degenerates into FLOSTFT. For real signals, when p=2, and for complex signals, when p=1, FLOASCT degenerates into SBCT. Therefore, FLOASCT is an extension of SBCT and represents a generalized method of SBCT.

#### 3.2.2. Application Review

In this section, the test signal consisting of two components is used to evaluate the performance of the proposed FLOASCT and the exiting SBCT methods, which is given as

(28) $$s(t)=\sin\left[2\pi (50t+10\sin(2t))\right]+\sin\left[2\pi (60t+10\sin(2t))t\right]=s_{1}(t)+s_{2}(t)$$

**Figure 9:**
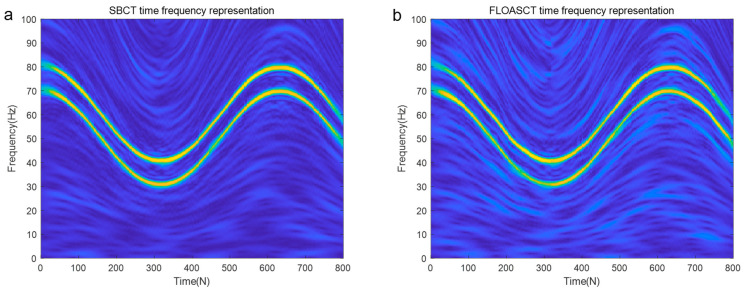
TFR of s(t) under Gaussian noise environment (α=2, SNR=8 dB). ((**a**). SBCT method; (**b**). FLOASCT method).

**Figure 10:**
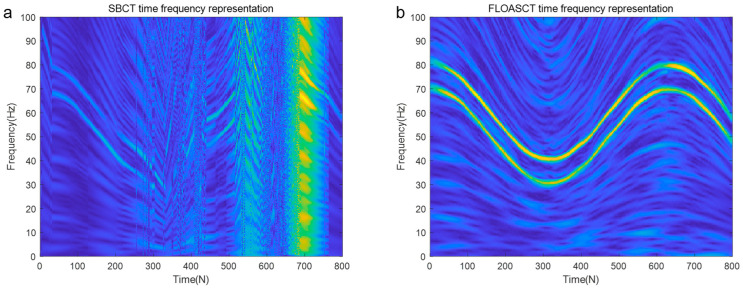
TFR of s(t) in infinite variance process noise environment (α=0.8, MSNR=18 dB). ((**a**). SBCT method; (**b**). FLOASCT method).

The sampling frequency of the signal s(t) is set to 200 hz, and its instantaneous frequency trajectory, which is a nonlinear function of time, can be repressed as f(t)=(50+20cos(2t))+(60+20cos(2t)), where $s_{1}(t)$ is with IF law 50+20cos(2t), and IF of $s_{2}(t)$ is 60+20cos(2t). The FLOASCT and SBCT time frequency representation methods are applied to reveal a nonlinear change in the frequency of the signal s(t) with time. The experiments are conducted with infinite variance process noise and Gaussian noise separately, and the results are shown in Figure 9 and Figure 10.

To better test and indicate the rotating angles generating ability of the TF basis employing the exiting SBCT method and the proposed FLOASCT algorithm, the test signal consisting of two components is used to evaluate their performance, which is given as follows:

(29) $$s(t)=\sin(\int_{-\infty }^{+\infty }2\pi f_{basis1}(t)dt)+\sin(\int_{-\infty }^{+\infty }2\pi f_{basis1}(t)dt)=s_{1}(t)+s_{2}(t)$$

**Figure 11:**
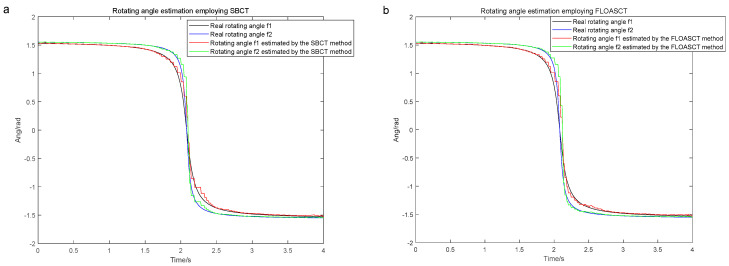
The real rotating angles $\theta_{1}$ and $\theta_{2}$ of the TF basis and the angles of inclination ${\hat{\theta }}_{1}$, ${\hat{\theta }}_{2}$ under Gaussian noise environment (α=2, SNR=8 dB). ((**a**). SBCT method; (**b**). FLOASCT method).

**Figure 12:**
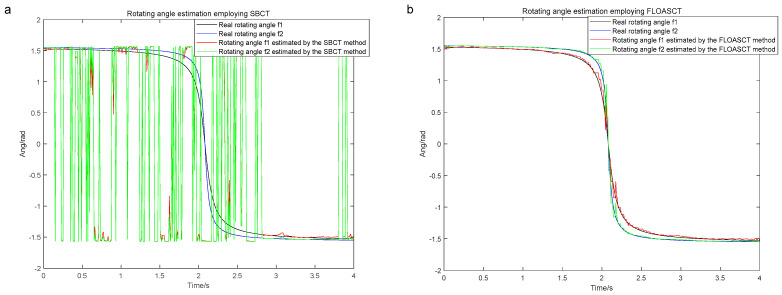
The real rotating angles $\theta_{1}$ and $\theta_{2}$ of the TF basis and the angles of inclination ${\hat{\theta }}_{1}$, ${\hat{\theta }}_{2}$ in infinite variance process noise environment (α=0.8, MSNR=18 dB). ((**a**). SBCT method; (**b**). FLOASCT method).

The sampling frequency of the signal s(t) is set to 200 hz, and its base frequency trajectory $f_{basis1}$ is set as $f_{basis1}=-6t^{2}+25t+5$, and $f_{basis2}$ is with IF law $f_{basis2}=-12t^{2}+50t+10$. The FLOASCT and SBCT methods are applied to compare the real rotating angles $\theta_{1}$ and $\theta_{2}$ of the TF basis with the angles of inclination ${\hat{\theta }}_{1}$, ${\hat{\theta }}_{2}$ generated by them and evaluate the performance. The comparison experiments with the SBCT and FLOASCT methods are conducted with infinite variance process noise and Gaussian noise separately, and the results are shown in Figure 11 and Figure 12, respectively.

**Figure 13:**
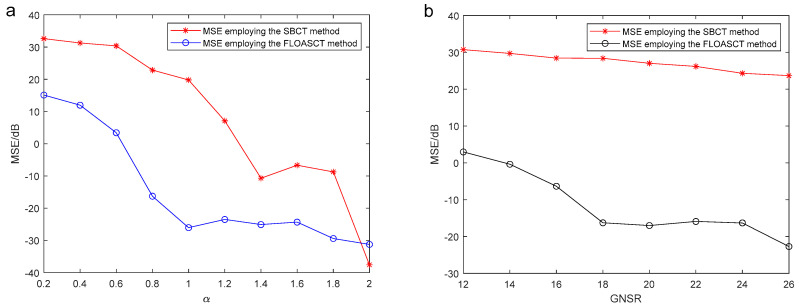
MSE of the rotating angles $\theta_{1}$ and $\theta_{2}$ and angle of inclination ${\hat{\theta }}_{1}$, ${\hat{\theta }}_{2}$ generated by the SBCT and FLOASCT methods. (**a**) The experiment in different characteristic index α (0.2–2) when MSNR=18 dB; (**b**) the experiment in different MSNR (12 dB–26 dB) when α=0.8.

In the following, we select s(t) as the test signal. We give the SBCT and FLOASCT methods applied in analyzing MSE of the real rotating angles $\theta_{1}$ and $\theta_{2}$ of the TF basis and the angles of inclination ${\hat{\theta }}_{1}$ and ${\hat{\theta }}_{2}$ generated by them under different characteristic indexes α and MSNR. The simulation result is shown in Figure 13. The mean square error (MSE) of the real $\theta_{1}$, $\theta_{2}$ and the angles of inclination ${\hat{\theta }}_{1}$, ${\hat{\theta }}_{2}$ employing the SBCT and FLOASCT methods can be written by

(30) $$MSE=10\log_{10}\left\{\frac{1}{K}\sum_{n=1}^{2}{\left[{\hat{\theta }}_{n}-\theta_{n}\right]}^{2}\right\}$$

where K is the number of the Monte-Carlo experiment. The experiments with the SBCT and FLOASCT methods are conducted with infinite variance process noise and Gaussian noise separately, and the results are shown in Figure 13.

#### 3.2.3. Remarks

Figure 9 shows the analysis results using the SBCT and FLOASCT methods in a Gaussian environment (α=2, SNR=8 dB), where both methods clearly depict the two IF trajectories in their TFRs. The TFR results of the signal using the SBCT and FLOASCT methods are shown in Figure 10 in an infinite variance process noise environment (α=0.8, MSNR=18 dB). As illustrated, the SBCT method fails to accurately provide the IF trajectory of the signal s(t), as shown in Figure 10a. Furthermore, it is challenging to demonstrate the TFR of the signal in such specific noise conditions. However, the proposed FLOASCT method demonstrates its advantage in resisting infinite variance process noise, still clearly representing the signal’s time-frequency distribution.

Figure 11a,b show the changing curves of the real rotating angles $\theta_{1}$ and $\theta_{2}$ of the TF basis and the angles of inclination $\theta_{1}$ and $\theta_{2}$ using the SBCT and FLOASCT methods, respectively, in a Gaussian noise environment (α=2, SNR=8 dB). The results indicate that the rotation angles of the TF bases generated by the two methods are approximately equal to the tilt angles simultaneously produced by all IF trajectories at all time centers. In contrast, as shown in Figure 12a, the angles of inclination $\theta_{1}$ and $\theta_{2}$ generated by the SBCT deviate from the true rotation angles in an infinite variance process noise environment (α=0.8, MSNR=18 dB). However, the tilt angle generated by the FLOASCT method is still approximately equal to the actual rotation angle of the TF basis, as shown in Figure 11b, which proves the accuracy advantage of the proposed method.

Figure 13a is the SBCT and FLOASCT methods’ MSE of the rotating angles $\theta_{1}$ and $\theta_{2}$ and angle of inclination ${\hat{\theta }}_{1}$, ${\hat{\theta }}_{2}$ generated by the SBCT and FLOASCT methods when characteristic index α changes from 0.2 to 2 and MSNR=18 dB in an infinite variance process noise environment. It shows that when α<2, the MSE obtained using the proposed FLOASCT method is always lower than the MSE obtained by the exiting SBCT method. And two methods’ MSE variation of the real $\theta_{1}$, $\theta_{2}$ and the generated ${\hat{\theta }}_{1}$, ${\hat{\theta }}_{2}$ is given in Figure 13b when MSNR goes from 12 dB to 26 dB and α=0.8. The results show that when MSNR changes, MSE varies only slightly. The angles of inclination ${\hat{\theta }}_{1}$, ${\hat{\theta }}_{2}$ generated by the FLOASCT method are approximately equal to the actual rotation angles of the TF basis, whereas the MSE obtained using the SBCT method exhibits larger deviations. The above experiments demonstrate that the proposed algorithm can achieve better performance in the Gaussian noise or infinite variance process noise environment, whether in the case of low feature index or low signal-to-noise ratio, reflecting its resilience and advantages.

## 4. Application Simulations

In this section, the performance of the proposed FLOALCT and FLOASCT methods is validated using real outer race fault signals obtained from the Case Western Reserve University Bearing Data Center [37]. The parameters selected for the experiment include a sampling frequency of 12,000 Hz, a fault size of 0.021 inches, and a motor speed of 1797 revolutions per minute (rpm), with the outer race position centered at 6:00 relative to the load zone. We intercepted the signals of 3600 points contaminated by the noise of the infinite variance process as the experimental signals.

**Figure 14:**
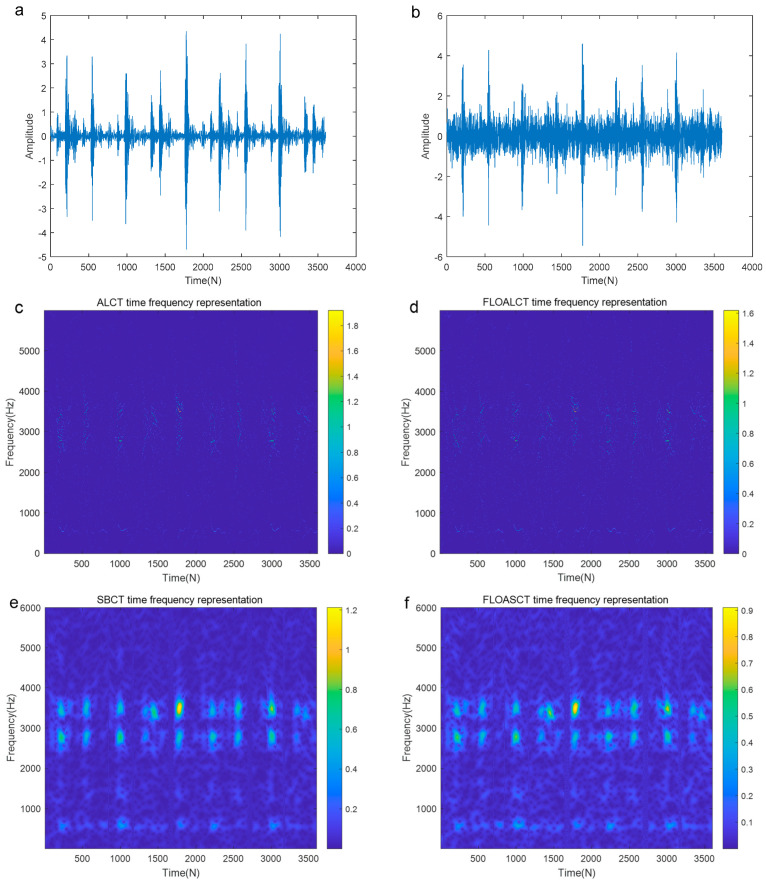
TFR of the outer race fault signal contaminated by Gaussian noise (α=2, SNR=3 dB). ((**a**). The original signal; (**b**). The signal contaminated by Gaussian noise (α=2, SNR=3 dB); (**c**). ALCT TFR; (**d**). FLOALCT TFR; (**e**). SBCT TFR; (**f**). FLOASCT TFR).

**Figure 15:**
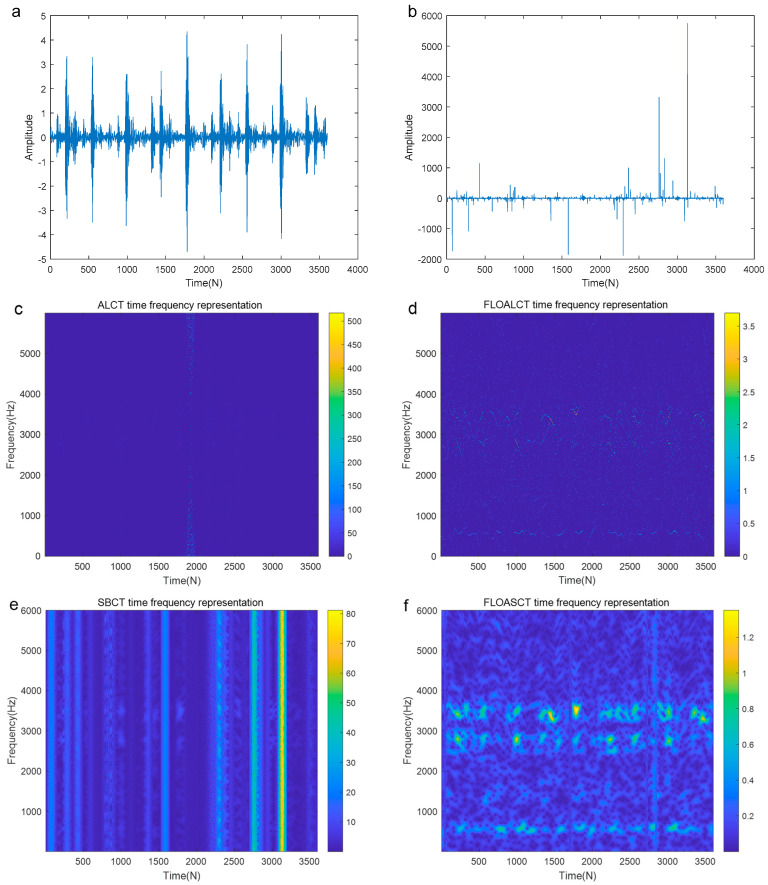
TFR of the outer race fault signal contaminated by infinite variance process noise environment (α=0.8, MSNR=18 dB). ((**a**). The original signal; (**b**). The signal contaminated by infinite variance process noise (α=0.8, MSNR=18 dB); (**c**). ALCT TFR; (**d**). FLOALCT TFR; (**e**). SBCT TFR; (**f**). FLOASCT TFR).

The origianl signal is demonstrated in Figure 14a and Figure 15a and the signal contaminated by Gaussian noise (α=2, SNR=3 dB) and those contaminated by α stable distribution noise (α=0.8, MSNR=18 dB) are shown in Figure 14b and Figure 15b, respectively. It can be seen that the signal is completely submerged by the low MSNR α stable distribution noise. Figure 14c,e display the Time-Frequency Representation (TFR) of the real outer race fault signal contaminated by infinite variance process noise (α=2, SNR=3 dB) using the existing ALCT and SBCT methods, respectively. The corresponding TFRs obtained using the proposed FLOALCT and FLOASCT methods are shown in Figure 14d and Figure 14f, respectively. All four methods successfully identify the physically meaningful characteristic frequency trajectories of the fault signal. From the figures, it can be observed that the mechanical fault signal exhibits regular vibrations with a time interval of approximately 33 milliseconds between components, indicating a characteristic frequency of about 30 Hz. Additionally, the TFR plots reveal that the fault signal’s vibration components correspond to three frequency components: 600 Hz, 2800 Hz, and 3500 Hz.

**Figure 16:**
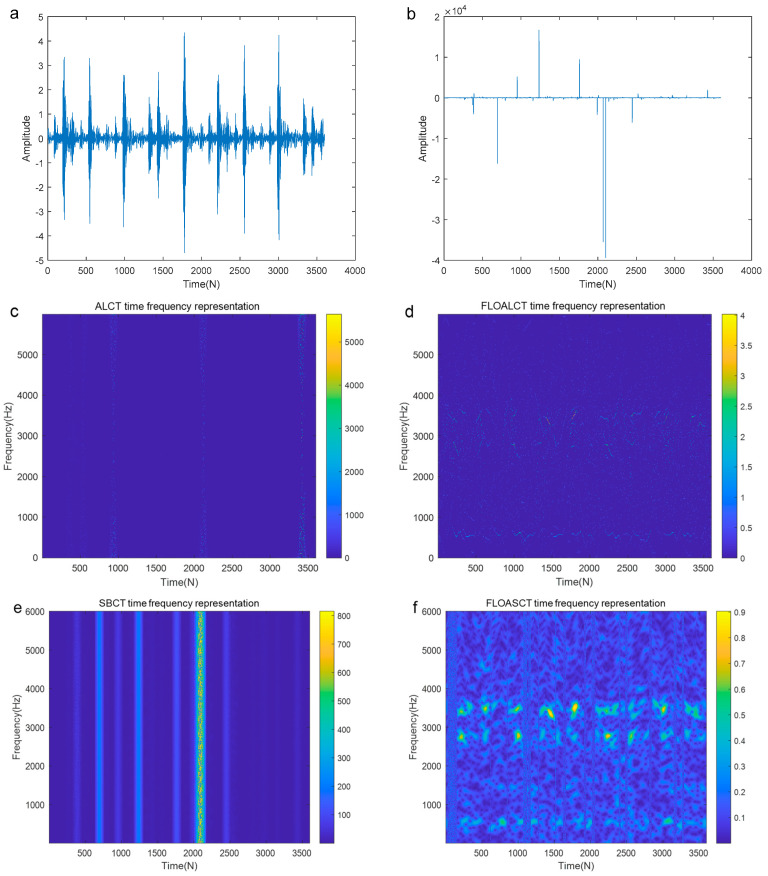
TFR of the outer race fault signal contaminated by infinite variance process noise environment (α=0.8, MSNR=8 dB). ((**a**). The original signal; (**b**). The signal contaminated by infinite variance process noise (α=0.8, MSNR=8 dB); (**c**). ALCT TFR; (**d**). FLOALCT TFR; (**e**). SBCT TFR; (**f**). FLOASCT TFR).

Figure 15c,e present the TFR of the fault signal polluted by infinite variance process noise (α=0.8, MSNR=18 dB) using the ALCT and SBCT methods, respectively. The results indicate that these two methods fail to recognize the characteristic information of the fault signal. However, as seen in Figure 15d,f, the proposed methods still clearly display the frequency variation information of the fault signal regularity, demonstrating their adaptive capabilities. In order to further verify the performance of the improved methods under low MSNR conditions, we have added the experimental simulations of the bearing outer race fault signal with MSNR=8 dB and α=0.8, and the result is shown in Figure 16a–f. Compared with the original signal in Figure 16a, Gaussian environment in Figure 14b, and high MSNR in Figure 15b, due to the lower MSNR, the signal in Figure 16b is more contaminated and the noise pulse is stronger. The ALCT and FLOASCT methods in Figure 16c,e are powerless, but the FLOALCT and FLOASCT methods are still effective under the environment.

**Figure 17:**
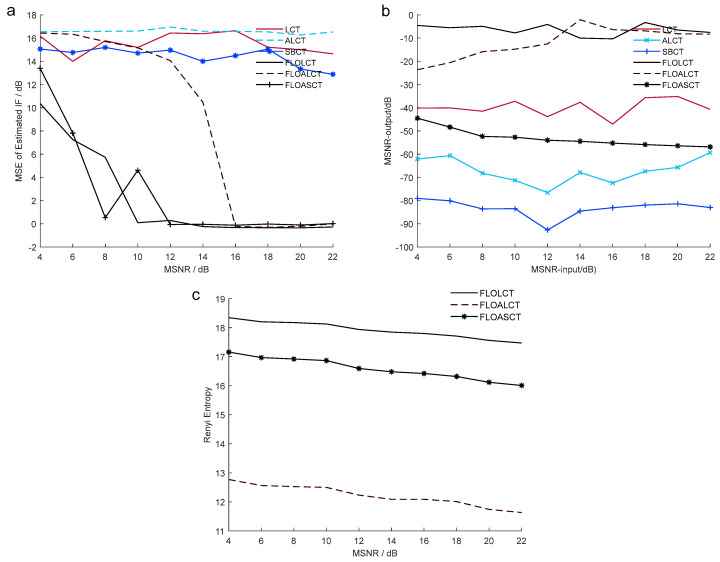
(**a**). MSE of IF estimation employing the LCT, ALCT, SBCT, FLOLCT, FLOALCT, and FLOASCT methods in different MSNR settings; (**b**). MSNR-output of the LCT, ALCT, SBCT, FLOLCT, FLOALCT, and FLOASCT methods in different MSNR settings; (**c**). Renyi entropy of the improved FLOLCT, FLOALCT, and FLOASCT methods in different MSNR settings (4–22 dB) when α=0.8.

**Figure 18:**
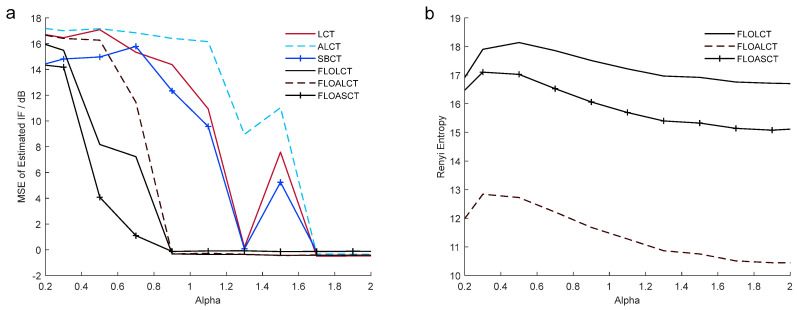
(**a**). MSE of IF estimation employing the LCT, ALCT, SBCT, FLOLCT, FLOALCT, and FLOASCT methods in different (0.2–2) when MSNR=18 dB; (**b**). Renyi entropy of the improved FLOLCT, FLOALCT, and FLOASCT methods in different α.

To further investigate the impact of MSNR and the characteristic exponent α on the proposed FLOALCT and FLOASCT methods, we compare their performance with LCT, ALCT, SBCT, and FLOCLT methods under various MSNR and α levels by presenting their MSE, MSNR-output, and Renyi entropy results in Figure 17 and Figure 18. Figure 17a illustrates that FLOALCT and FLOASCT exhibit lower MSE in IF estimation than ALCT and SBCT. Their MSNR-output is higher than existing methods, demonstrating a stronger capability to resist infinite variance process noise, as shown in Figure 17b. Compared with the existing FLOLCT methods, they have higher time-frequency aggregation capabilities, and the energy concentration of the TFR obtained using the FLOALCT method in Figure 15c is the best.

Figure 18a shows that when the noise’s characteristic index α>0.9, the MSE of the two proposed methods is approximately equal to 0. Compared with the existing methods, they have lower IF estimation errors and are more stable. It can be seen in Figure 16b that the FLOALCT method consistently achieves the highest time-frequency concentration when α varies from 0.2 to 2. The time-frequency concentration capability of the FLOASCT method is higher than that of FLOCLT, indicating that the proposed methods offer better signal time-frequency resolution than existing methods. These experimental results demonstrate that the proposed FLOALCT and FLOASCT methods provide better time-frequency concentration and robustness in infinite variance process noise of different MSNR values and characteristic exponent α.

To evaluate the computational efficiency of robust FLOALCT and FLOASCT, we compared their computational time with other methods. The result is given in Table 2. The system configuration of the computer used in the test process was Intel (R) Core (TM) i7-10700 CPU @ 2.90 GHz, 16.0 GB RAM, 1 TGB SSD, and the software used was MATLAB R2020b. It can be seen from the figure that the calculation times of FLOALCT and FLOASCT are close to those of ALCT and SBCT, respectively. They require more time than FLOSTFT and FLOLCT, but the corresponding TFR has a higher energy concentration. In most practical applications, the computational efficiency of FLOALCT and FLOASCT should be acceptable.

The FLOALCT method employs an adaptive iterative process involving signal amplitude ranking, extraction, elimination, and re-ranking to sequentially extract all signal components, which are subsequently aggregated to reconstruct TFR. This approach enhances SNR and yields a TFR with superior time-frequency concentration compared to FLOLCT. Particularly noteworthy is its enhanced multi-component analysis capability, which demonstrates exceptional performance when processing multi-component signals with intersecting frequency trajectories.

The FLOASCT method replaces the conventional kernel function in FLOLCT with a scaled kernel phase function, which adaptively adjusts the chirp rate by scaling the time center point and its surrounding time-frequency basis functions according to frequency and time variations. This method can provide a high-resolution time-frequency representation of multi-component signals with short frequency intervals and strong background noise without obtaining prior knowledge in advance. FLOASCT can precisely and adaptively match the slope angles of the IF ridges of multi-component signals. However, there is a local ambiguity phenomenon in its time-frequency representation. The local peaks of each time-frequency point of the signal are the largest at the frequency center and gradually decrease to both sides within the window range.

FLOSTFT offers low time-frequency resolution but has a simple algorithmic structure and is suitable for preliminary analysis of the mechanical fault signals. FLOLCT enhances FLOSTFT by introducing a chirp rate parameter, which improves energy concentration for linear frequency-modulated signals. However, its fixed chirp rate limits its applicability in mechanical fault diagnosis, as it fails to achieve high energy concentration at each time instant. FLOALCT is particularly effective for analyzing multi-component fault signals with crossing frequency trajectories in the time-frequency domain. FLOASCT performs well in processing the nonlinear frequency-modulated multi-component signals, especially those with closely spaced frequencies and strong background noise. A comprehensive comparison of the features, limitations, and application scenarios of the proposed FLOALCT and FLOASCT TFR methods, along with the existing fractional lower-order cumulant-based methods (FLOSTFT and FLOLCT), is summarized in Table 3. In the application analysis of actual mechanical fault signals, the optimal method should be selected based on specific fault characteristics, such as background noise intensity, multi-component nature, and presence of frequency crossings. Choosing the appropriate method according to these criteria will yield more accurate and reliable analysis results.

## 5. Conclusions

In this study, we proposed two robust time-frequency analysis methods called FLOALCT and FLOASCT, which extend FLOLCT and FLOSTFT, employing fractional low-order statistical moments. The FLOALCT method enhances noise immunity and improves time-frequency concentration by sequentially extracting and reconstructing signal components based on their energy distribution in the FLOLCT time-frequency domain. Meanwhile, FLOASCT employs an adaptive scaling mechanism that dynamically adjusts both the time center point and time-frequency basis functions to optimally match IF ridge curvature, enabling precise tracking of nonlinear frequency variations. This approach is particularly effective for obtaining high-resolution time-frequency representations under strong background noise conditions. Comparison results with the existing TFA methods show that the proposed FLOALCT and FLOASCT can achieve superior time-frequency energy concentration representation, lower Renyi entropy values, smaller MSE of IF and larger MSNR-output values in different MSNR settings and α. The advantages in all aspects demonstrate that the proposed algorithm has stronger adaptive ability and better performance. The results in the application of the actual mechanical outer race fault signals also demonstrate their excellent feature extraction and analysis capabilities.

## Figures and Tables

**Table 1:** The novel statistical model parameters of the normal and fault signals.

Type	Infinite Variance Process Model Parameters			
	α	β	γ	μ
Normal signal	2	0.3328	0.1097	−0.0072
Inner race fault	1.6646	0.0976	0.1185	−0.0127
Rolling element fault	1.5985	0.1968	0.1170	−0.0025
Outer race fault	1.4639	0.0036	0.0900	−0.0064

**Table 2:** Total computing time of the methods.

Methods	FLOSTFT	FLOLCT	ALCT	FLOALCT	SBCT	FLOASCT
Computing Time (s)	0.0835	0.1314	1.2164	1.5217	3.8218	3.8424

**Table 3:** The features, deficiencies, and application scenarios of the improved FLO adaptive CT frequency representation methods.

Methods	Features	Deficiencies	Application Scenarios
FLOSTFT	Simple algorithm	Low time-frequency resolution	Early analysis for the mechanical fault signals
FLOLCT	High energy concentration cannot be achieved at every point in time	Constant chirp rate	Suitable for linear fault signal analysis
FLOALCT	Strong anti-interference ability, high time-frequency concentration	It has local time-frequency diffusion	Multicomponent fault signals with cross frequency trajectories
FLOASCT	High time-frequency concentration and strong adaptive ability without prior conditions	TFR indicates the existence of local blurring	Multicomponent fault signal with close frequency interval and large background noise

## Data Availability

The authors confirm that the data supporting the findings of this study are available within the article.
